# Large-scale climatic effects on traditional Hawaiian fishpond aquaculture

**DOI:** 10.1371/journal.pone.0187951

**Published:** 2017-11-16

**Authors:** Daniel McCoy, Margaret A. McManus, Keliʻiahonui Kotubetey, Angela Hiʻilei Kawelo, Charles Young, Brandon D’Andrea, Kathleen C. Ruttenberg, Rosanna ʻAnolani Alegado

**Affiliations:** 1 Department of Oceanography, University of Hawaiʻi Mānoa, Honolulu, Hawaiʻi, United States of America; 2 Paepae o Heʻeia, Kāneʻohe, Hawaiʻi, United States of America; 3 Department of Geology and Geophysics, University of Hawaiʻi Mānoa, Honolulu, Hawaiʻi, United States of America; 4 Sea Grant College Program, University of Hawaiʻi Mānoa, Honolulu, Hawaiʻi, United States of America; Centro de Investigacion Cientifica y de Educacion Superior de Ensenada Division de Fisica Aplicada, MEXICO

## Abstract

Aquaculture accounts for almost one-half of global fish consumption. Understanding the regional impact of climate fluctuations on aquaculture production thus is critical for the sustainability of this crucial food resource. The objective of this work was to understand the role of climate fluctuations and climate change in subtropical coastal estuarine environments within the context of aquaculture practices in Heʻeia Fishpond, Oʻahu Island, Hawaiʻi. To the best of our knowledge, this was the first study of climate effects on traditional aquaculture systems in the Hawaiian Islands. Data from adjacent weather stations were analyzed together with *in situ* water quality instrument deployments spanning a 12-year period (November 2004 –November 2016). We found correlations between two periods with extremely high fish mortality at Heʻeia Fishpond (May and October 2009) and slackening trade winds in the week preceding each mortality event, as well as surface water temperatures elevated 2–3°C higher than the background periods (March-December 2009). We posit that the lack of trade wind-driven surface water mixing enhanced surface heating and stratification of the water column, leading to hypoxic conditions and stress on fish populations, which had limited ability to move within net pen enclosures. Elevated water temperature and interruption of trade winds previously have been linked to the onset of El Niño in Hawaiʻi. Our results provide empirical evidence regarding El Niño effects on the coastal ocean, which can inform resource management efforts about potential impact of climate variation on aquaculture production. Finally, we provide recommendations for reducing the impact of warming events on fishponds, as these events are predicted to increase in magnitude and frequency as a consequence of global warming.

## Introduction

Over 400 million people depend upon fish protein as part of their daily diet [1]. While the yield of global capture fisheries has remained static since the 1950s due to overexploitation, the contribution of aquaculture is increasing dramatically, from 3.9% in 1970 to 36% in 2006 [2], finally overtaking wild-caught fish in 2014 [3]. As our dependence on aquaculture grows, improving the resilience of aquaculture systems with respect to climate fluctuation and climate change is critical. Here, we consider the regional influence of a dynamic global climate mode on traditional Hawaiian aquaculture systems.

The El Niño Southern Oscillation (ENSO) is a climate mode in which variations in sea surface temperature (SST) in the Eastern and Central Equatorial Pacific Ocean alter global ocean currents and atmospheric circulation patterns [4,5]. Under neutral conditions, consistent trade winds along the equator confine warm equatorial sea-surface waters to the western Pacific, forming a persistent ‘warm pool’. During the evolution of an El Niño event, weakening equatorial trade winds cause a reversal in the atmospheric pressure gradient between the western and eastern Pacific, culminating in gradual movement of the warm pool eastward across the Pacific. Globally, this shift in equatorial conditions accounts for the main sources of regional year-to-year variability in weather and climate [6,7]. Climate change is predicted to alter the amplitude, frequency, seasonal timing, and spatial patterns of ENSO events [8–10], such as the occurrence of the Central Pacific El Niño (El Niño Modoki), distinguished by anomalous warming in the Central Pacific and cooling in the Eastern and Western Equatorial Pacific.

By virtue of its sub-tropical location, Hawaiʻi experiences relatively minimal seasonal variation in incoming solar radiation [11]. The surrounding ocean provides a buffer against significant changes in SST, and a consistent northeast trade wind belt produces a robust orographic rain cycle on the windward side of each island [12]. From October-April, stochastic storm events originate from the Northeast Pacific basin approximately 2,000 miles away, and tend to interrupt the trade winds. Elevated SST around Hawaiʻi is only weakly correlated with the onset of traditional El Niño events [13]. Instead, El Niño events typically displace the subtropical jet stream, weakening trade winds, leading to decreased precipitation in winter and slightly enhanced rainfall in summer [14,15]. In contrast to conventional El Niño evolution, central equatorial warm SST anomalies associated with El Niño Modoki extend northeastward toward Hawai`i, eventually reaching the west coast of North America, a unique feature not regularly associated with traditional El Niño [16]. Importantly, El Niño Modoki events have evolved up to 3 times more frequently since 1990 than traditional El Niño events, exhibiting warmer SST anomalies in the North Pacific than their pre-1990 counterparts [17]. Therefore, Modoki events likely have had significant influence on recent Hawaiian SST patterns [16].

**Fig 1:**
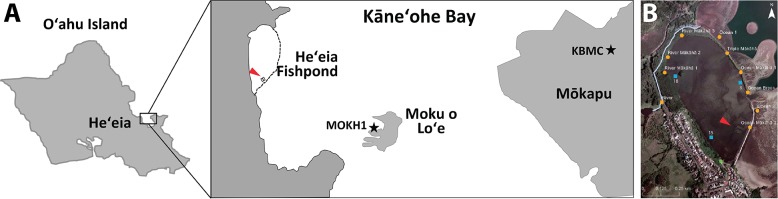
Study site. A) Map of Oʻahu, Hawai‘i with locations of He‘eia Fishpond, the National Buoy Data Center station ‘MOKH1’, and the Kāne‘ohe Bay Marine Core Base Air Station (KBMCB). B) Heʻeia Fishpond stations used in this study (map downloaded from USGS National Map viewer). Orange circles = mākāhā, blue squares = *in situ* TidbiT^®^ sites, green pentagon = air temperature reference, red arrowhead = net pen enclosures.

As one of only 14 fishponds (loko iʻa [Hawaiian language]) remaining in production across the state of Hawaiʻi, Heʻeia Fishpond on the windward coast of Oʻahu Island serves as a model for Native Hawaiian community-based, regional aquaculture. It also serves as a long-term field site for understanding the effects of urbanization on coastal ecosystems. Conservative estimates place the number of loko iʻa in the Hawaiian Islands at ~488 in 1778 (establishment of sustained contact with Europeans), with the potential to produce at least 900 metric tons of fish per year [18,19]. The prospect of revitalizing these culturally and economically significant sites for sustainable aquaculture emphasizes the need to re-evaluate these systems within the context of climate change. In this study, academic scientists (University of Hawaiʻi Mānoa) and a community organization (Paepae o Heʻeia, POH) collaborated to investigate the effect of the 2009 El Niño on fish species reared in Heʻeia Fishpond by loko iʻa practitioners. From May 24—May 29, 2009 nearly 3,000 Pacific threadfin (*Polydactylus sexfilis*, moi [Hawaiian language]) died within the constructed net pens of He’eia Fishpond (Fig 1). We describe these massive fish mortality events as ‘fish kills’. Subsequently, a second fish kill on October 10, 2009 eliminated the remaining stock of 10,000 moi. As a result of these mortality events, Paepae o Heʻeia halted use of net pens in order to focus on habitat restoration, thereby significantly decreasing total aquaculture production.

These two fish kill events in Heʻeia Fishpond occurred at the onset of the 2009–2010 El Niño Modoki [7]. Given the short time scale over which the fish kill events occurred, it seemed likely that both fish kills resulted from short-term climate-induced environmental stress, symptomatic of a classic marine heatwave [20]. We hypothesize that the 2009–2010 El Niño Modoki contributed to conditions that culminated in both fish mortality events within Heʻeia Fishpond. To understand the environmental conditions surrounding both fish kills, water temperature data over a 10-month time frame (March-December 2009) taken from *in situ* loko iʻa sensors were compared to historic climate observations (wind, air temperature, rainfall, and water temperature) spanning twelve years: November 20, 2004—November 20, 2016. Increased water temperature and interrupted trade winds associated with the 2009–2010 El Niño Modoki coincided with both Heʻeia Fishpond fish mortality events. We hypothesize that abrupt, short-term variability in water temperature and wind fields resulting from ENSO events can act as drivers of fish kills in these environments. Our hypothesis is consistent with numerous studies across the globe, linking increased mean temperatures to changes to marine ecosystems [20,21]. These studies become even more critical given the prediction that extreme climate events will increase in frequency and magnitude as a result of climate change [9,22,23].

## Methods

### Study site

Heʻeia Fishpond (21.437111°N, 157.810214°W) is a 0.356 km^2^ (88 acre) loko iʻa located in Kāneʻohe Bay within the district of Koʻolaupoko on Oʻahu (Fig 1A). The fishpond was estimated to have been constructed approximately 800 years ago [24]. The fishpond is entirely enclosed by a 2.5 km wall (kuapā [Hawaiian language]) composed of fossilized coral and basalt with sluice gates (mākāhā [Hawaiian language]) allowing flow of sea water into and out of the embayment. In addition, an irrigation ditch (auwai [Hawaiian language]) running parallel to the western kuapā, as well as submarine groundwater sources, supply freshwater from Heʻeia stream to the fishpond to maximize primary production of micro- and macro-algae, traditional food sources for target herbivore fish species [24]. Each mākāhā is a flume with a horizontal floor and vertical basalt rock or concrete mortar walls (concrete mortar walls were added in the last 50 years), typically with one m^2^ dimensions [25]. Two of the freshwater mākāhā were placed above the normal tidal range to encourage unidirectional flow of Heʻeia Stream tributaries into Heʻeia Fishpond, and a third mākāhā was positioned adjacent to Heʻeia Stream, allowing bi-directional flow based on the semi-diurnal tidal cycle [25].

Though historically productive, land-use changes in the Heʻeia watershed over the last century have altered the normal functioning of Heʻeia Fishpond. In particular sediment loading from Heʻeia Stream due to agriculture and urbanization has overwhelmed the original mechanisms by which material is advected out of the pond, resulting in progressive accumulation of terrigenous particulates on the coral benthos. Today, the fishpond is roughly 1 m deep [25]. In addition, major flood events destroyed sections of the kuapā. In 1927, pressure from Heʻeia Stream caused a large section of kuapā abutting the southernmost freshwater mākāhā to burst, transforming that area of the fishpond into a “diffuse flow region” that has never been repaired [25]. Residual parts of the wall formed an island to the southeast of the area [24]. In 1965, rapid flooding of the fishpond after heavy rains led to a 50 m break in the eastern ocean kuapā (“Ocean Break”, OB). The break in the kuapā was not fully repaired until 2015 and instead was replaced by a 0.9 m high, 79 m long retaining wall composed of concrete cylinders that elbowed into the pond. The elbow at the OB retained a minimum volume of water within the fishpond but was lower than the height of the surrounding intact kuapā (1.20 m) such that water overflowed during spring tides [25].

Since 2001, the Kamehameha Schools has leased Heʻeia Fishpond to POH, a non-profit Native Hawaiian community organization dedicated to restoring the fishpond to its original form and revitalizing aquaculture in accordance with traditional native Hawaiian practices. Quarter-acre fish pens in the southeastern sector of the loko iʻa, totaling one acre (Fig 1B), were used for research and education from 2001–2006, and then prioritized for aquaculture of Pacific threadfin (moi) starting in 2006. From 2006–2009, Heʻeia Fishpond produced approximately 1.2 metric tons of moi, consistent with historical productivity of Native Hawaiian fishponds [18]. Typically, moi were spawned at the Oceanic Institute (Waimanalo, HI) and transferred at between 60–90 days of age to net pens in Heʻeia Fishpond (Fig 1B). Fish were reared on Skretting’s Marine Grower^TM^ (43% crude protein, 14% crude fat, 2.0% crude fiber, 1.3% phosphate), and were typically grown until they reached 400 g or 15 months of age. At the time of the fish kill, the net pens were stocked at a density of 16,000 fish per acre, and fish were 8 months old (May 2009) and 13 months old (October 2009), respectively.

### Nā Kilo Honua o Heʻeia coastal ocean observing system

Since 2007, Nā Kilo Honua o Heʻeia (www.nakilohonuaoheeia.org) has quantified the spatial and temporal variability of the chemical and physical properties of Heʻeia Fishpond through monthly sampling of the surface and benthic water column at a network of sites throughout the interior and exterior of the loko iʻa [25]. This study was conducted with the permission of Kamehameha Schools (Joey Char, Land Asset Manager, Kamehameha Schools Community Engagement and Resources Division). *In situ* HOBO^®^ water-level data loggers (Onset Computer Corp, Bourne, MA) were periodically deployed on either side of the mākāhā to record water level and temperature, and TidbiT^®^ v2 temperature sensors (Onset Computer Corp, Bourne, MA) were deployed at selected stations to establish a temperature time series and spatial distribution [25,26]. Sensors were placed 20 cm above the seabed and were programmed to collect data at 10-min intervals 3-months at a time. Sensors were encased in perforated PVC pipe to prevent animal interference. Data were downloaded before the sensors were cleaned, reformatted and redeployed. Proximal water temperature and water level data were obtained from a sensor on the interior of the kuapā ocean break and a sensor placed on the interior of the second freshwater mākāhā (‘OB’ and ‘River Mākāhā 2’, Fig 1C). An air temperature reference (‘Air Ref’, Fig 1C) was secured ~2 m above the ground in the southwest corner of the fishpond. Internal loko iʻa temperature prior to and after each fish kill were evaluated from three consecutive deployment periods of TidbiT^®^ and HOBO^®^ sensors from 2009. Specifically, data from instruments deployed at stakes 13, 15, and 18 (Fig 1B) were used since these stations were continuously monitored over the entire period of interest. Before deployment, the temperature sensors were programmed to sample from the same water bath. The variation between instruments was less than +/-0.1 degree. For the purposes of our analysis, the data record was treated as continuous (March 1 –December 1, 2009), as the sensors were removed between deployments for only 72–96 hours. All data processing was performed off-line using MATLAB^®^ (The MathWorks Inc., Natick MA, 2008b).

### Physical observations

Flow direction, water level, and water velocity measurements were taken in each mākāhā using a SonTek Argonaut Shallow Water (SW) Profiler (Xylem Inc., San Diego, CA) at a frequency of 1 minute for an interval of 10 seconds. Since water column height ranged from 15–60 cm in each mākāhā the measured cell size was set to 5 cm with a blanking distance of 10 cm. The accuracy of this instrument is reported by the manufacturer to be ±1% of the measured velocity. The Argonaut SW Profiler and battery housing were attached to a 60 cm^2^ plastic platform that was secured to a 2.5 cm steel plate. The instrument package was deployed at the central point of the mākāhā floor, centered between the mākāhā walls and the interior/exterior edge of the mākāhā. Due to its size and three-dimensional flow, the OB was sampled using a NorTek Aquadopp profiler (Nortek USA, Boston, MA), at a sampling frequency of 1 minute with an interval of 10 seconds. The water column in OB ranges from 150–250 cm, therefore the measured cell size was set to 10 cm with a blanking distance of 20 cm. The Aquadopp Profiler was secured to a cinder block via two hose clamps, and deployed in the middle of OB with two 6.8 kg weights attached to the bottom of the cinder block and buried in the sediment to prevent the instrument package from varying from its vertical orientation. Water velocity measurements (m/sec) were taken by the SonTek and the NorTek current meters, and multiplied by the area (m^2^) of the water column within the mākāhā to produce water volume flux values (m^3^/sec). Continuous depth measurements from the SonTek and NorTek were used to compensate for the change in area (Δ m^2^/sec) between tidal states. Current velocity measurements were taken independently in each mākāhā for a period of one week, and all mākāhā were sampled between 21 January and 11 May 2012. To quantify fresh and salt water flux within the fishpond, flow rates through each mākāhā and the Ocean Break were measured (Fig 1B, Table 1). Mean flux during spring and ebb tides from the oceanic mākāhā (OM1, OM2, OB, TM), which border Kāneʻohe Bay, suggest that the pond is more influenced by oceanic inputs (>94% of total mean flux) than freshwater inputs (<6% of total mean flux) from Heʻeia Stream (Table 1).

**Table 1:** Mean and maximum water volume flux (m^3^/sec) for each mākāhā during flood and ebb tidal states [26].

Site	Mean (m^3^/sec)		Max (m^3^/sec)		Percent of total mean (%)	
	Flood	Ebb	Flood	Ebb	Flood	Ebb
Ocean Mākāhā 1	1.5918	0.8272	3.9137	1.8195	56.43	29.29
Ocean Mākāhā 2	0.2834	0.1449	0.5776	0.4074	10.05	5.13
Ocean Break	0.1585	0.1675	0.3891	0.3112	5.62	5.93
Triple Mākāhā	0.6378	1.5245	1.3547	2.8144	22.73	53.99
River Mākāhā 2	0.0634	0.0803	0.2170	0.2634	2.25	2.84
River Mākāhā 3	0.0855	0.0795	0.2182	0.2649	3.05	2.82

Historical air temperature, wind direction, wind speed, and precipitation data from November 2004—November 2016 were obtained from a MesoWest station [27] at the Kāne’ohe Bay Marine Corps Air Base (KBMCB) and the NOAA National Data Buoy Center (MOKH1 http://www.ndbc.noaa.gov/station_history.php?station=mokh1); locations shown in Fig 1A. Station KBMCB is located on the Mōkapu Peninsula, roughly 3 km northeast of Heʻeia Fishpond at 4.8 m elevation. Buoy MOKH1–1612480 –Moku o Loʻe, HI (21.432°N 157.790°W), located 1 km southeast of Heʻeia Fishpond, has fixed sensors to record air temperature 2.83 m above the kuapā, wind direction and wind speed 10 m above the kuapā, and a water temperature sensor 1.23 m below the sea surface at mean low low water (MLLW). We used MOKH1 water temperature as a proxy for Kāneʻohe Bay water temperature. Data taken from the MOKH1 buoy extends from November 2008 –November 2016 (NBDC 2017).

To identify El Niño and La Niña events from 2004–2016, the Oceanic Niño Index [28] was applied to meteorological data between May and October of each respective calendar year within this period. Warm, cold and neutral phases of ENSO were compared to determine whether the environmental conditions present during both fish kills were influenced by the onset of the 2009 El Niño Modoki.

## Results and discussion

### Fishpond characteristics: Flow and climate variability

**Fig 2:**
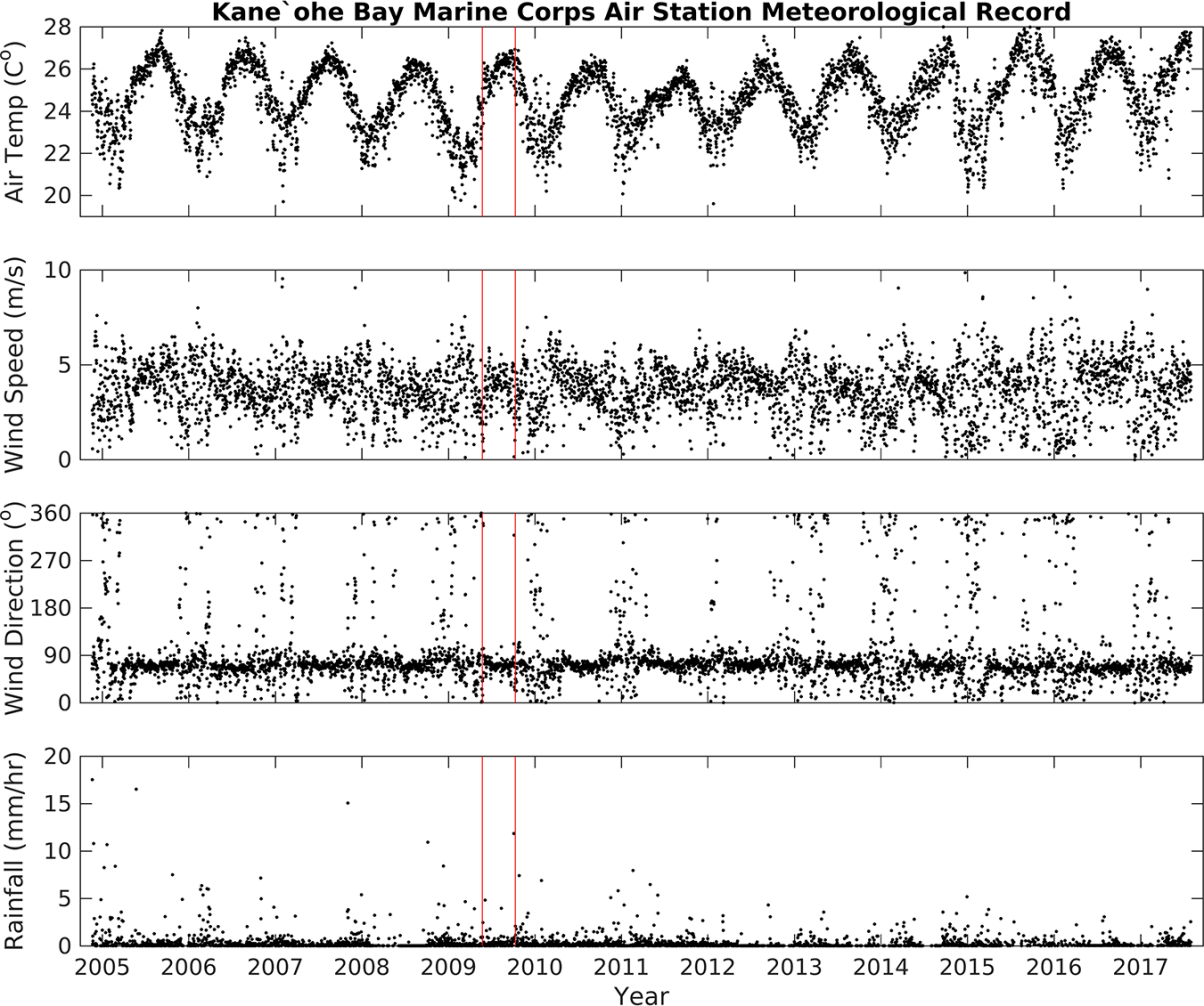
Time series of air temperature, wind speed, wind direction, and rainfall data from the Kāne‘ohe Bay Marine Core Base Air Station, November 20, 2004 –November 20, 2016. Red vertical lines = approximate fish kill dates.

**Fig 3:**
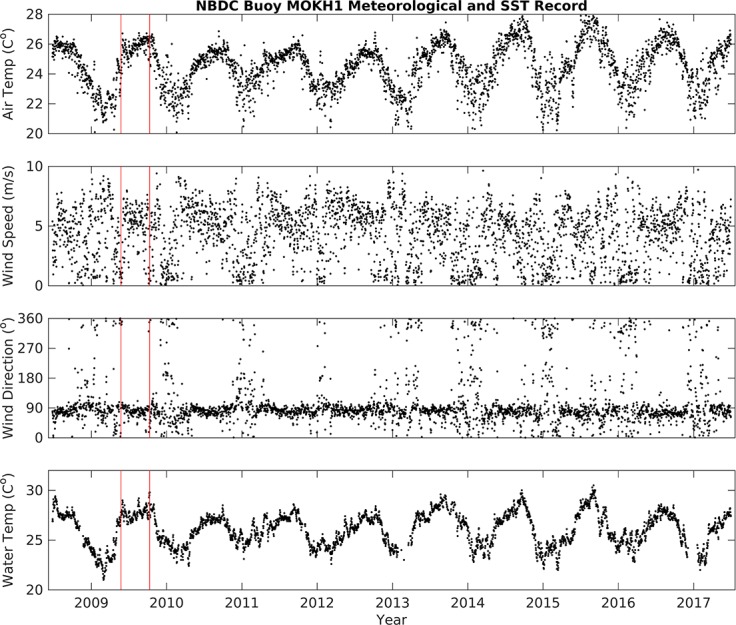
Time-series of air temperature, sea surface temperature, wind direction, and wind speed data from the National Buoy Data Center station ‘MOKH1’, November 20, 2008 –November 20, 2016. Red vertical lines = approximate fish kill dates.

**Fig 4:**
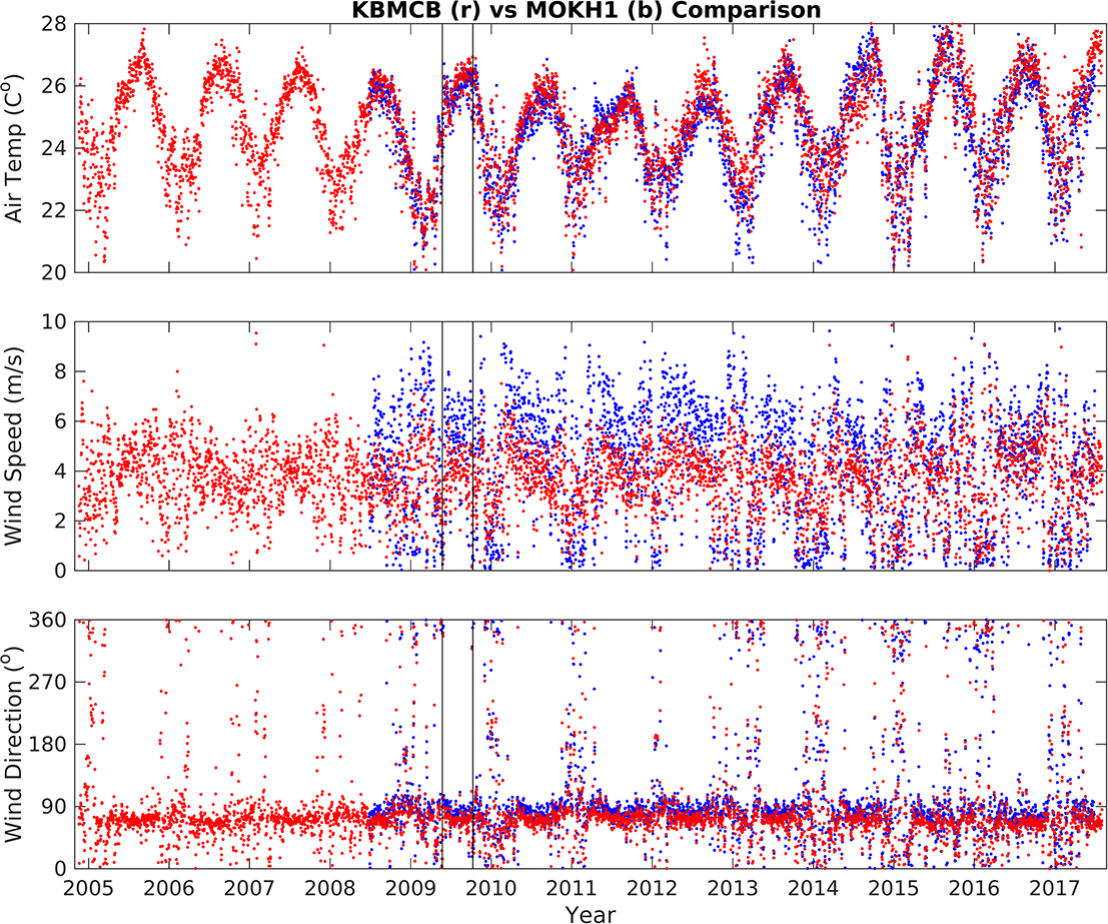
Comparison of daily averages of air temperature (°C), wind speed (m/s), and wind direction (°) data from Kāne‘ohe Bay Marine Core Base Air Station (KBMCB) and MOKH1 buoy. Black vertical lines = approximate dates of the fish kills.

The long-term variability of air temperature, wind speed, and rainfall data from KBMCB and water temperature from MOKH1 was estimated from daily averages (Figs 2 and 3). Twelve years of data (November 2004 –November 2016) reveal: air temperature averaged 24.6 ± 2.1°C, wind speed and direction averaged 3.7 m/s and 67° (respectively) with periods of 0 m/s wind speeds accounting for 14% of the time series (Table 2), and precipitation averaged 0.64 ± 3.69 mm/hr. The records of air temperature, wind speed, and wind direction measurements from MOKH1 and KBMCB were consistent for these same periods (Fig 4). The high standard deviation for precipitation indicates variable and inconsistent amounts of rainfall. Water temperature data for Kāneʻohe Bay from November 2008 –November 2016 (MOKH1 water temperature) averaged 26.10°C with a standard deviation of ±1.67°C.

**Table 2:** Background meteorological conditions of Kāneʻohe Bay (observed at KBMCB, November 20^th^ 2004-November 20^th^ 2016) as compared to conditions during the fish mortality events (May and October 2009).

	Background Nov 2004—Nov 2016	May Fish Kill May 17–24, 2009	October Fish Kill Oct 3–10, 2009
Wind Speed (m/s)	3.7 ± 1.9	2.8 ± 2.3	1.8 ± 1.7
Wind Direction (°)	67	5	112
% NaN^a^	14%	33%	44%
Rainfall (cm/hr.)	0.64 ± 3.69	0.03 ± 0.05	0.22 ± 0.33
Air Temp (°C)			
Kāneʻohe Bay	24.6 ± 2.1	23.2 ± 2.0	26.1 ± 1.8
Heʻeia Fishpond^b^	-	22.8 ± 3.5	25.8 ± 3.6

^a^ Anemometer registered NaN or not-a-number under conditions of 0 m/s wind speed; frequency of no wind (% NaN) is reported here.

^b^ Fishpond air temperature during fish mortality events was measured with Air Ref HOBO logger, Fig 1.

### Elevated water temperature preceded fish mortality events

**Fig 5:**
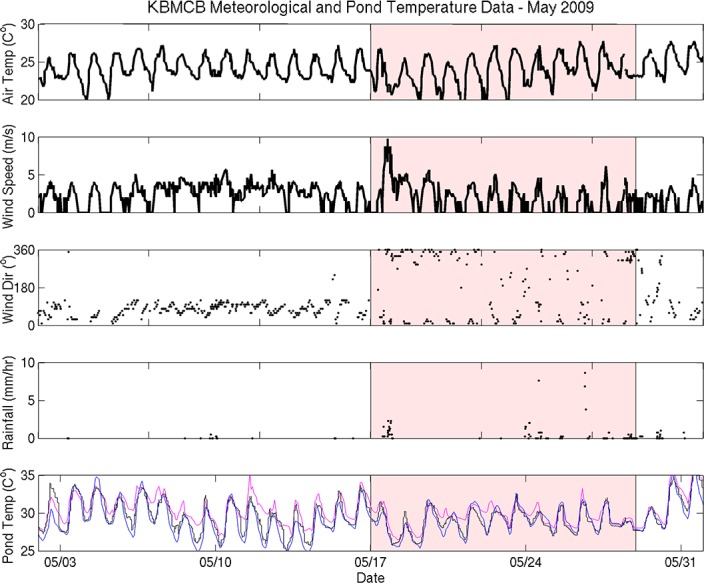
Time-series of Kāne‘ohe Bay Marine Core Base Air Station (KBMCB) data, May 2009. The week before the fish kill was analyzed separately (shaded red). Fishpond temperatures from each station are as follows: Stake 13 = blue, Stake 15 = black, Stake 18 = pink.

**Fig 6:**
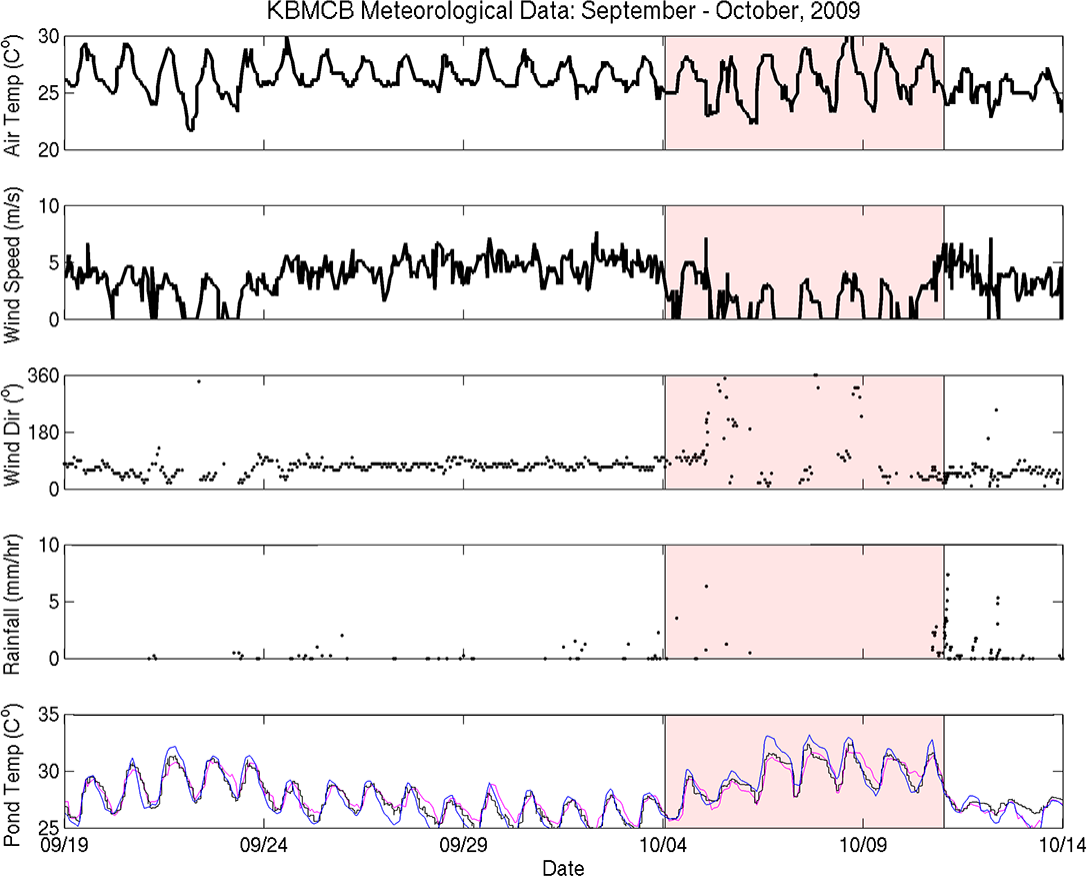
Time-series of Kāne‘ohe Bay Marine Core Base Air Station (KBMCB) data from September 15^th^–October 15^th^, 2009. The week before the fish kill was analyzed separately and is shaded in red. Fishpond temperatures from each station are as follows: Stake 13 = blue, Stake 15 = black, Stake 18 = pink.

We observed a small upward trend in decadal water temperatures in Kāneʻohe Bay (Fig 3), however we found that this trend alone was not significant to trigger mortality events in Heʻeia Fishpond. Among the possible catalysts triggering fish mortality events, we considered short-term variability, spanning days to a year. Data from the week prior to each fish kill was compared against long-term means from the 2004–2016 time-series. The periods of time one week prior to each fish kill (May 17–29 and October 3–10, 2009) were analyzed statistically and compared to background conditions from March to December 2009. Mean air temperatures in Kāneʻohe Bay and the fishpond the week prior to the May fish kill, 23.3 ± 2.0°C and 22.8 ± 3.5°C respectively (Table 2), were cooler than the 2004–2016 mean of 24.6 ± 2.1°C (Table 2), while the week prior to the October fish kill was slightly higher than the mean in both Kāneʻohe Bay and the fishpond, 26.1 ± 1.8°C and 25.8 ± 3.6°C, respectively (Table 2). The daily air temperature range in Kāneʻohe Bay began to rise 5 days prior to each fish kill event, after May 17^th^ 2009 and after October 4^th^ 2009 (Figs 5 and 6, top panel). Despite a larger range in air temperature values, deviation from the background mean was not significant. We therefore excluded changes in air temperature as a cause of the fish kills. Rainfall data were also not significantly different from background trends. The average rainfall from May 17–24 2009 was 0.03 ± 0.05 cm/hr, while the average rainfall from October 3–10 was higher, at 0.22 ± 0.33 cm/hr. (Table 2), with a storm event (defined as >5 cm in a 24 hr period, or 0.2083 cm/hr. [29]) on October 4, 2009 (Fig 6). Despite the October storm event, there was no significant increase or decrease in rainfall prior to the fish kills.

Mean water temperature data from the MOKH1 in Kāneʻohe Bay were compared with *in situ* pond temperature data from instruments deployed between March–December 2009. Mean water temperatures in Kāneʻohe Bay were not significantly different from the mean water temperature from March-December 2009 (26.5 ± 2.0°C) during the week prior to the May and October fish kills (27.0 ± 0.5°C and 28.4 ± 0.9°C, respectively) (Table 3). In contrast, water temperatures from fresh water and oceanic inputs to Heʻeia fishpond were higher in the week prior to each fish kill (Table 3). The mean temperature at River Mākāhā 2 in May 17–24, 2009 was 3.6°C higher and 1.9°C higher in October 3–10, 2009 than baseline conditions (25.6 ± 3.1°C from March–December 2009). Likewise, the mean temperature at Ocean Break in May 17–24, 2009 was 1.6°C higher and 3°C higher in October 3–10, 2009 than baseline (25.7 ± 2.7°C from March–December 2009). Finally, the mean water column temperature within the fishpond at all three sampling sites in Heʻeia fishpond (Stakes 13, 15, and 18) was elevated the week prior to each fish kill, as compared to March-December 2009, 26.7 ± 2.9°C, 26.9 ± 2.8°C, and 27.1 ± 2.9°C, respectively (Table 3). From May 17–24 2009 the mean temperatures at Stake 13 and 15 were 2.0°C higher than baseline and 2.5°C higher at Stake 18. In October 3–10 2009, the mean temperature Stake 13 was 3.2°C higher, 2.6°C higher at Stake 15, and 2.4°C higher at Stake 18 than baseline.

**Table 3:** Mean and standard deviation water temperature (°C) from the fishpond and coastal ocean (MOKH1 surface buoy) over 3 different periods: March–December 2009, May 17^th^– 24^th^ 2009, and October 3^rd^–October 10^th^ 2009.

	Mar–Dec 2009	May 17–24 2009	Oct 3–10 2009
MOKH1	26.5 ± 2.0	27.0 ± 0.5	28.4 ± 0.9
Stake 13	26.7 ± 2.9	28.7 ± 1.8	29.9 ± 2.0
Stake 15	26.9 ± 2.8	28.9 ± 1.5	29.5 ± 1.6
Stake 18	27.1 ± 2.9	29.6 ± 1.4	29.5 ± 1.6
Ocean Break	25.7 ± 2.7	27.3 ± 1.2	28.7 ± 1.7
River Mākāhā 2	25.6 ± 3.1	29.2 ± 1.8	27.5 ± 1.8

Fishpond water sensors were placed 20 cm above the sediment water interface at Stakes 13, 15, and 18, River Mākāhā 2, Ocean Break [25].

**Fig 7:**
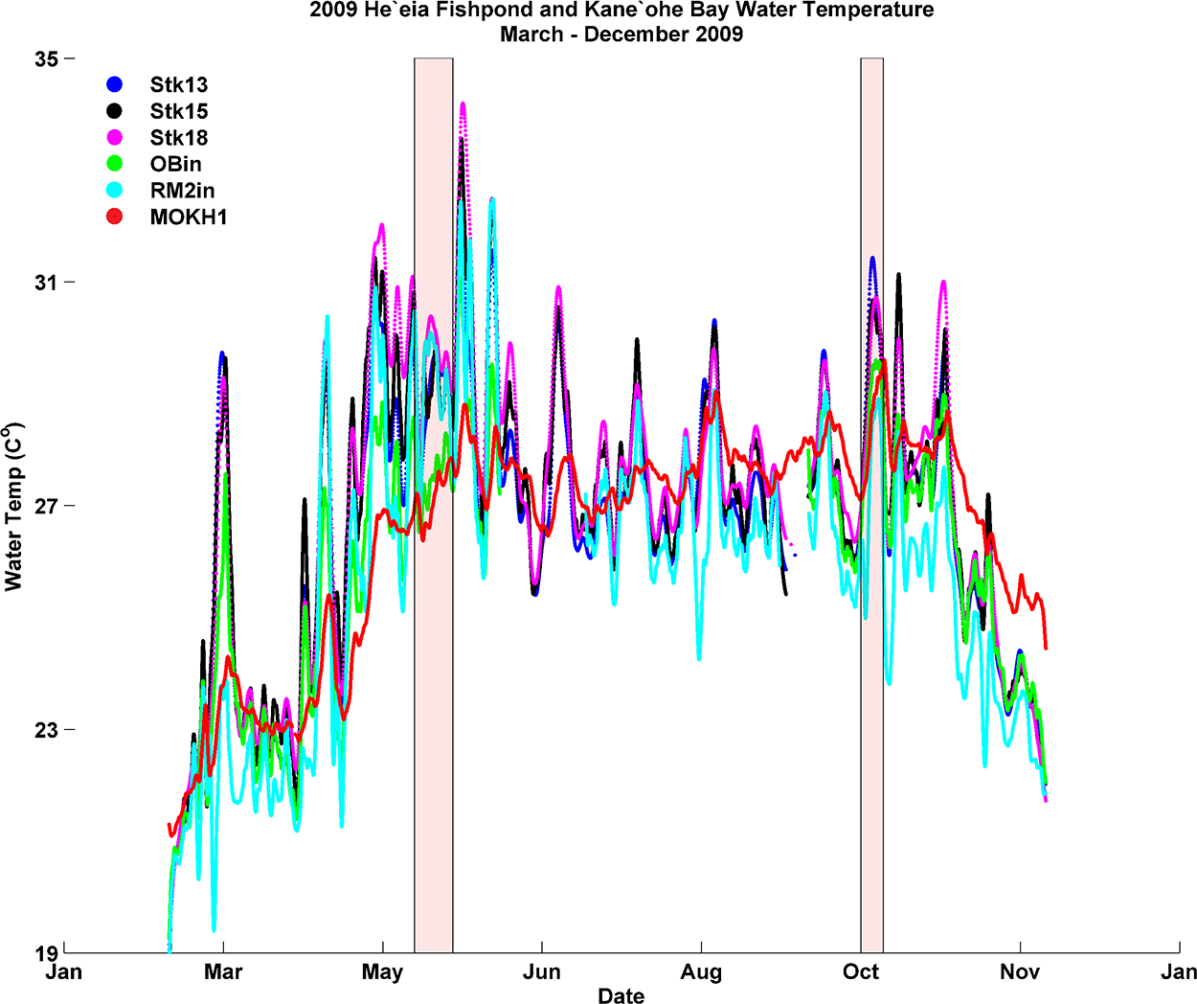
Time-series of He‘eia Fishpond and Kāne‘ohe Bay water temperature (cubic spline fit; see text) from March to December 2009. Red shaded areas = approximate dates of the fish kills.

Water temperature from all stations generally remained between 25°C—29°C from May to late October 2009, after which time temperatures dropped to near 23°C (Fig 7. The *in situ* data record over approximately 6 months represents a significant period of time over which inner and outer fishpond water temperature (River Mākāhā 2, Ocean Break, MOKH1) experienced consistently warmer temperatures relative to the mean temperatures from the March-December 2009 time frame (Table 3).

Mortality rates of fish typically increase with abnormally high water temperatures; some species do not feed when thermally stressed, while others have a greater demand for energy for digestion, growth, muscle performance, and metabolism [21,30]. No formal thermal tolerance curve for adult moi exists, however the thermal tolerance of laboratory-reared eggs and larvae is between 21.9–28°C [31] and 25–27°C for nursery-stage development of moi [32]. Our data suggest that loko iʻa water temperatures exceeded ideal temperature ranges for moi particularly during peak solar radiation hours. During both mortality events, mean water temperature reached nearly 30°C, with maximum daily temperatures approaching 35°C. Moreover, most estuarine fish species are unable to regulate their body temperature and typically seek thermal refuge within their habitat to escape colder or warmer pools within the host water body [30]. Vulnerability of estuarine species to temperature changes is enhanced in shallow water habitats where daytime temperatures may reach the upper thermal limits tolerable by fish [30]. Thus, the rise in summer water temperatures in and around He’eia Fishpond, with even higher spikes during the weeks preceding each kill, may have been a factor in the fish kills.

### Anomalous wind patterns

Previous studies have shown lack of wind for extended periods to be a factor in stratification of shallow ponds [33,34]. Even in estuaries as shallow as Heʻeia Fishpond (1–2 m), daytime stratification is enhanced during periods of low to no winds [35]. Without mixing of the water column through wind forcing a thermally driven density gradient can form. As oxygen is produced in the surface seawater via photosynthesis and evasion from the atmosphere, depletion of oxygen is simultaneously observed at the sediment water interface (SWI) due to consumption via microbial respiration of organic matter [35]. Thus, in addition to lack of ventilation via wind mixing, we hypothesize that long periods of calm winds can result in hypoxia (concentrations of dissolved oxygen less than 2 mg/L) in shallow water bodies, conditions associated with large-scale fish kills [35].

Heʻeia Fishpond stewards maintain daily records of environmental conditions and fish behavior. An entry in the loko iʻa steward log during the May 2009 mortality event described “depressed feeding behavior by fish within the pens”, and in October 2009 loko iʻa practitioners again noted an “absence of trade winds” and “hot, muggy weather,” prefacing a die off of the entire fish population within the pens. To assess the entries in the log, the wind record was examined within the context of the temperature time-series to determine if changes in wind patterns influenced the fishpond.

The 2009 mean wind direction and speed was 3.5 m/s from a direction of 69°, reflecting typical trade winds, whereas wind patterns during each fish kill were significantly different (Table 2, Figs 5 and 6). From May 17 –May 24 wind speeds were depressed (2.8 m/s), shifted to mean direction of 5°, and 33% of the directional record is Not-A-Number (NaN), indicating that wind speeds dropped to zero, a condition in which directional data cannot be recorded. A two-fold increase in days with zero wind from May 17–24 is significant, since over the summer of 2009 only 16% of the wind direction record was NaN. From October 4 –October 11, average winds were 1.8 m/s from 112°, and 44% of the wind direction data record were NaN, or below detection (Table 2).

**Fig 8:**
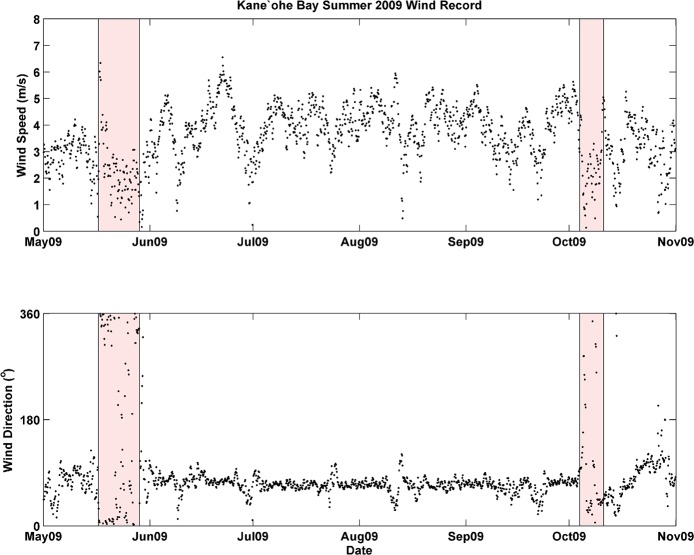
Time series of wind direction and wind speed (3 hr averages) from KBMCB, May—November 2009.

Wind data spanning May-November 2009 (Fig 8) were consistent with analyses of the period prior to and during each fish kill (Table 2). Wind speed and wind direction were nearly uniform from June to October, exhibiting typical trade wind values (~5.0–6.0 m/s from ~70–80°), with infrequent interruptions. This pattern was significantly altered, however, on two separate occasions when wind speed dropped to near zero over the span of several days. The timing of these low/no-wind events overlaps with the dates of each fish kill.

While our data suggest a link between decreased winds, hypoxia and the fish mortality events observed in Heʻeia Fishpond, *in situ* dissolved oxygen data were not available to confirm a correlation of this parameter with wind events. It is clear, however, that suppression of the trade winds for a weeklong period during both the May and October fish kills contributed to the altered physical properties of Heʻeia Fishpond. Moreover, the shallow depth of the fishpond may have promoted thermal stress via tidal dynamics; both May and October fish kills occurred during the new moon lunar cycle, when the system experiences extremely low tides. The periodically reduced depth of the water column would have increased water heating time and limited fish movement (vertically) within the net pens. In addition, the net pens were located next to OM2, the mākāhā with the least tidal flux, in the southern region of the fishpond (Fig 1, Table 1).

### Wind and SST correlations with 2009 El Niño

Previous studies have indicated that ENSO events may influence SST and wind patterns in the vicinity of the Hawaiian Islands [13,16]. To determine whether a link exists between the conditions experienced during each kill and the regional effect of the 2009 El Niño Modoki, data from KBMCB and MOKH1 were separated into summertime segments (May—October) and analyzed within the context of the respective ENSO climate mode occurring during that year. Four ENSO conditions were analyzed: the 2007 La Niña, the 2009 El Niño Modoki, the 2010 La Niña, and the 2015 El Niño [36], which was a canonical Eastern Pacific pattern. The 2012 and 2013 ENSO-neutral periods were also analyzed. With the onset of El Niño Modoki over the Pacific during early 2009, the possibility that ENSO conditions influenced both SST and wind patterns around O`ahu was evaluated using MOKH1and KBMCB data.

**Fig 9:**
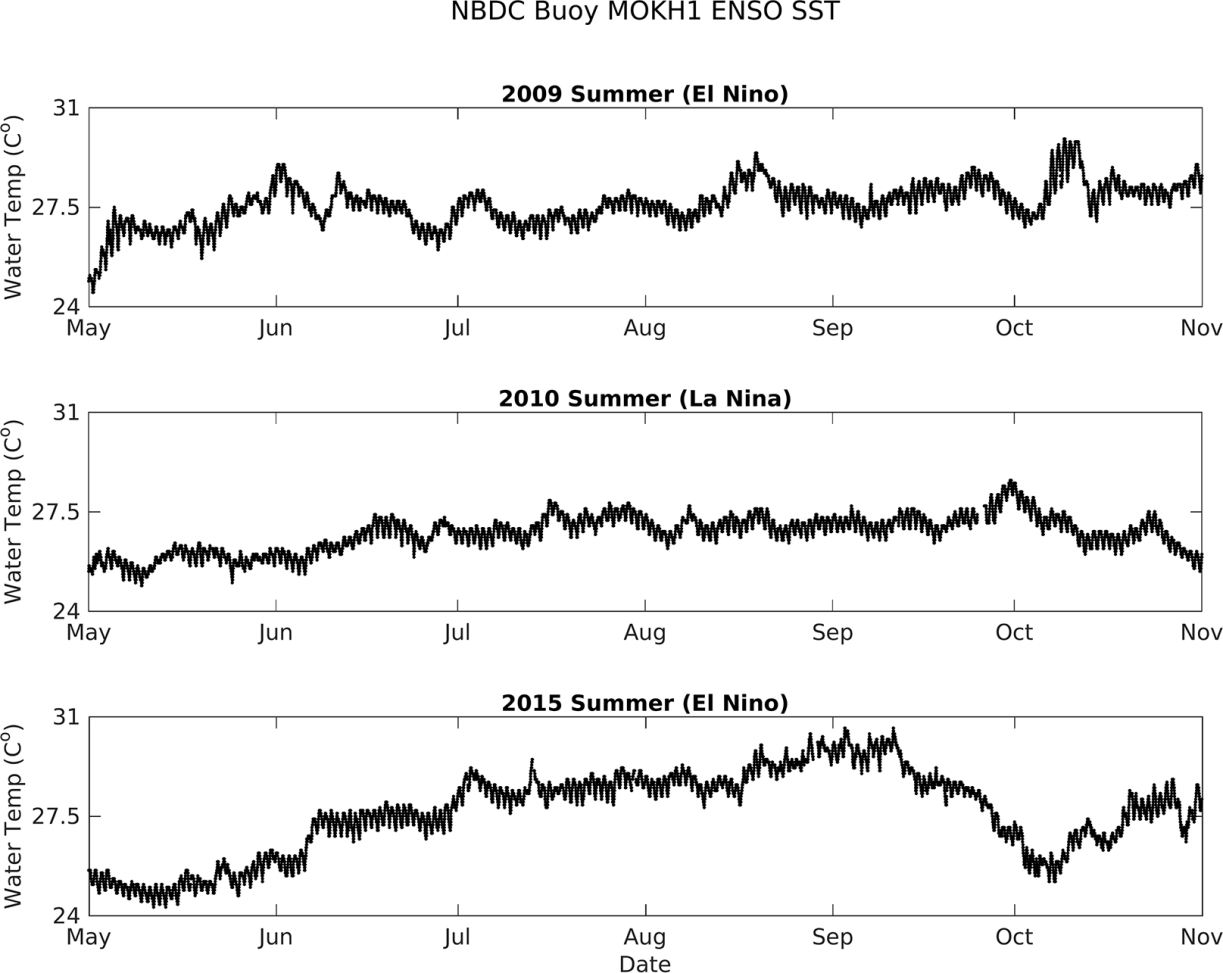
Kāne‘ohe Bay sea-surface temperature from MOKH1 from May–November 2009 (El Niño Modoki), 2010 (La Niña), and 2015 (El Niño).

Mean and standard deviation of air temperature, wind, rainfall, and water temperature data for each mode are reported in Table 4. Water temperature data were not available for the 2007 La Niña due to limitations of the MOKH1 time series (November 2008 –November 2016) and a gap of *in situ* instrument coverage inside the fishpond over this same period. Mean air temperature was highest during the 2015 El Niño and lowest in 2012, when ENSO-neutral conditions persisted. Mean water temperatures during the 2009 El Niño Modoki, the 2015 El Niño, and the 2013 ENSO-neutral period were ~1°C higher than during the 2010 La Niña and the 2012 ENSO-neutral events (respectively, Fig 9). The 2009 El Niño Modoki and the 2013 ENSO-neutral periods also exhibited depressed mean wind speeds and a higher percentage of no-wind periods in comparison to the other identified ENSO events. Rainfall mean and standard deviation was significantly greater during the 2009 El Niño Modoki and 2015 El Niño than both La Niña events and ENSO-neutral periods (Table 4).

**Table 4:** Mean and standard deviation of air temperature, wind speed, wind direction, and rainfall from KBMCB and water temperature from MOKH1 from May 1^st^—November 1^st^, 2007 (La Niña), 2009 (El Niño), 2010 (La Niña), 2012 (neutral), and 2013 (neutral), and 2015 (El Niño); n.d. = no data available.

	2007 La Niña	2009 El Niño	2010 La Niña	2012 Neutral	2013 Neutral	2015 El Niño
Wind Speed (m/s)	4.0 ± 1.4	3.5 ± 1.7	3.8 ± 1.4	3.9 ± 1.5	3.5 ± 1.6	4.4 ± 2.1
Wind Direction (°)	71	69	71	72	69	69
% NaN	9%	15%	8%	9%	14%	5%
Rainfall (cm/hr.)	0.15 ± 0.31	0.86 ± 2.58	0.32 ± 0.79	0.10 ± 0.61	0.22 ± 1.27	0.55 ± 1.51
Air Temp.	25.8 ± 1.4	25.6 ± 1.6	25.5 ± 1.4	25.3 ± 2.0	25.8 ± 2.1	26.0 ± 1.9
Water Temp.	n.d.	27.6 ± 0.7	26.7 ± 0.6	26.8 ± 0.8	28.0 ± 0.7	27.6 ± 1.5

*In situ* observations of both water temperature and wind speed/direction within Kāne’ohe Bay, Oʻahu suggest that Modoki El Niño events may negatively affect Hawaiian coastal fisheries [2,37]. Water temperatures within Heʻeia Fishpond rose sharply starting in May 2009 and persisted at elevated levels through the end of October 2009. Indeed, all input of water into Heʻeia Fishpond (data from OB, MOKH1 buoy, River Mākāhā data loggers) indicate that cooling from external fresh and oceanic sources was not possible, as mean SST of Kāneʻohe Bay was also elevated. Compared to mean water temperatures from the February to December 2009 deployment period, water temperatures from the River Mākāhā and the OB were significantly warmer (~2.0–4.0° C) during the week of both fish kills (Table 4).

Notably, periods of calm wind were more prevalent during the 2009 El Niño Modoki, suggesting that trade winds were suppressed compared to background mean wind patterns. Kāneʻohe Bay water temperature was also ~1.0 °C higher during the 2009 El Niño Modoki and the 2015 conventional El Niño than the 2010 La Niña and the 2012 ENSO-neutral period. This result is consistent with theories regarding El Niño Modoki as correlating positively with higher SST proximal to Hawai‘i [16], despite the highest summer water temperatures occurring in 2013. The anomalously high SST and lack of substantial winds observed during 2013 (Table 4) were attributed to uncharacteristic conditions in the North Pacific that developed into a large mass of relatively warm water associated with widespread mortality of fish and invertebrates. Substantial warming occurred in the region as a result of a persistent high-pressure system associated with anti-cyclonic circulation near the Gulf of Alaska, also led to weakened wind patterns [38]. However, no mortality events occurred in the fishpond during this period as loko iʻa practitioners had abandoned net pen production in favor of kuapā restoration that would enable utilization of the entire fishpond. Therefore, despite similar statistical observations with the 2013 ENSO-neutral period, environmental conditions specifically associated with the 2009 El Niño Modoki appear to have negatively influenced the conditions in Heʻeia Fishpond, eventually leading to the fish kills in May and October of that year.

## Conclusions and implications

For hundreds of years, the Hawaiian Islands supported a large and thriving populace by systematically creating sustainable agricultural systems spanning from ‘ridge to reef’ [39,40]. In addition to anticipating changes in anthropogenic influences on the surrounding area, Native Hawaiian resource management will need to anticipate and adapt to changing climate scenarios including localized changes to sea level, water temperature, salinity, and altered weather patterns. Importantly, traditional knowledge provides a historical baseline that, when integrated with contemporary scientific approaches, serves as a holistic framework to understand the consequences of climate variability and climate change for coastal ecosystems. The effect of multi-annual climate modes on aquaculture yield has not been extensively characterized, particularly in subtropical estuarine systems. Long-term monitoring data represents a critical component of developing effective management strategies for these communities. In this study, we also show a link between fish mortality events in a Hawaiian aquaculture system and extended periods of increased water temperatures and depressed trade winds, coincident with the 2009 El Niño. Taken together, our *in situ* instrument data and qualitative observational data from traditional practitioners enabled a retrospective analysis of fish mortality events in a subtropical coastal estuary. Our time series on the physical parameters of He'eia Fishpond is the basis for examining response of this loko iʻa to major climatic events over the past 12 years. Given the vulnerability of the fishery to low wind and marine heatwave events, we provide three recommendations to limit the mortality of moi in the system: (1) Moving net pens closer to the mākāhā with the highest flow rates (Ocean Mākāhā 1 and Triple Mākāhā) will serve to both decrease water temperatures and increase aeration in the pens, (2) the installation of artificial aeration systems in the pens to reduce hypoxia, and (3) implementing flexible harvest strategies at the onset of a warming event. Finally, applying a hierarchical approach to characterizing marine heatwave events [41], such as those caused by multi-annual climate patterns, may inform aquaculture policy and practice. Adaptive management in other regions that have experienced marine heatwaves, which may be appropriate to Hawaiʻi include identifying vulnerable temperature hot spots as well as early detection of species abundance changes [42]. An understanding of the impact of climate variability and climate change on the aquaculture initiatives of the Hawaiian Islands is critical for the sustainability of this important food resource.
